# MRI shows limited mixing between systemic and pulmonary circulations in foetal transposition of the great arteries: a potential cause of in utero pulmonary vascular disease

**DOI:** 10.1017/S1047951114000870

**Published:** 2014-06-16

**Authors:** Prashob Porayette, Joshua F.P. van Amerom, Shi-Joon Yoo, Edgar Jaeggi, Christopher K Macgowan, Mike Seed

**Affiliations:** 1 Department of Paediatrics, Hospital for Sick Children and University of Toronto, Toronto, Canada; 2 Department of Physiology & Experimental Medicine, Hospital for Sick Children and University of Toronto, Toronto, Canada; 3 Department of Diagnostic Imaging, Hospital for Sick Children and University of Toronto, Toronto, Canada

**Keywords:** Transposition of the great arteries, regional blood flow, magnetic resonance imaging, circulation

## Abstract

**Objectives:**

To investigate the relationship between foetal haemodynamics and postnatal clinical presentation in patients with transposition of the great arteries using phase-contrast cardiovascular magnetic resonance.

**Background:**

A severe and irreversible form of persistent pulmonary hypertension of the newborn occurs in up to 5% of patients with transposition and remains an important cause of morbidity and mortality in these infants. Restriction at the foramen ovale and ductus arteriosus has been identified as a risk factor for the development of pulmonary hypertension, and this can now be studied with magnetic resonance imaging using a new technique called metric optimised gating.

**Methods:**

Blood flow was measured in the major vessels of four foetuses with transposition with intact ventricular septum (gestational age range: 35–38 weeks) and compared with values from 12 normal foetuses (median gestational age: 37 weeks; range: 34–40 weeks).

**Results:**

We found significantly reduced flows in the ductus arteriosus (p<0.01) and foramen ovale (p=0.03) and increased combined ventricular output (p=0.01), ascending aortic (p=0.001), descending aortic (p=0.03), umbilical vein (p=0.03), and aorto-pulmonary collateral (p<0.001) flows in foetuses with transposition compared with normals. The foetus with the lowest foramen ovale shunt and highest aorto-pulmonary collateral flow developed fatal pulmonary vascular disease.

**Conclusions:**

We found limited mixing between the systemic and pulmonary circulations in a small group of late-gestation foetuses with transposition. We propose that the resulting hypoxia of the pulmonary circulation could be the driver behind increased aorto-pulmonary collateral flow and contribute to the development of pulmonary vascular disease in some foetuses with transposition.

Outcomes following the arterial switch operation for transposition of the great arteries are now excellent. However, up to 5% of patients with transposition are born with a severe and sometimes irreversible form of pulmonary vascular disease that can be fatal.^1^ Persistent pulmonary hypertension of the newborn remains an important cause of early morbidity and mortality in transposition.^2^ Prenatal constriction at the arterial duct and restriction at the oval foramen are associated with poor postnatal outcome and have been linked to the development of pulmonary vascular disease in utero in transposition,^3^ ^,^ ^4^ although the aetiology of pulmonary vascular disease in transposition remains unclear.^5^

Phase-contrast magnetic resonance imaging is the non-invasive gold standard for the quantification of blood flow in children with congenital heart disease.^6^ ^,^ ^7^ Our group has developed a technique to perform accurate phase-contrast magnetic resonance imaging (MRI) in the foetus called metric optimised gating.^8^ Metric optimised gating achieves high-resolution phase-contrast MRI without needing an electrocardiographic signal by obtaining temporally oversampled data and optimising for minimum artefact over a sequence of reconstructions at different heart rates. The technique has been validated against conventionally triggered phase-contrast flow measurements using an in vivo simulation of foetal vessels, comparing sequential measurements made in the neck vessels of exercising adult volunteers using both techniques.^9^ The feasibility of using phase-contrast MRI with metric optimised gating for human foetal blood flow quantification is described in our previous publication, detailing the distribution of blood flow in 12 late-gestation normal foetuses.^9^ More recently, the technique has been successfully applied to foetuses with left-sided congenital heart disease.^10^ Our aim was to use phase-contrast MRI with metric optimised gating to investigate the haemodynamic substrate for the development of pulmonary vascular disease in utero in foetuses with transposition.

## Materials and methods

### Foetal study

The SickKids research ethics board approved this study and informed consent was obtained for foetal MRI. A total of four consecutive pregnant mothers diagnosed with transposition with intact ventricular septum at our institution underwent MRI with phase-contrast cine imaging with metric optimised gating according to our previously published technique^9^ (foetal gestational age range: 35–38 weeks). We used a contemporary group of 12 normal foetuses as our control group (median gestational age: 37 weeks; range: 34–40 weeks).^9^ Flows were directly measured in the ascending aorta, descending aorta, main pulmonary artery, superior caval vein, umbilical vein, arterial duct, and right and left pulmonary arteries. Pulmonary blood flow was calculated as the sum of left and right pulmonary artery flow. Flow in the descending aorta at the diaphragm was assumed equal to flow in the suprahepatic inferior caval vein such that oval foramen flow was calculated by subtracting the venous return to the right ventricle, which is the sum of the superior caval vein and descending aortic flows, from the right ventricular output. The right ventricular output is the flow in the great vessel connected to the right ventricle, which is the ascending aorta in transposition, or main pulmonary artery in normals. Aorto-pulmonary collateral flow was estimated by subtracting descending aortic flow at the diaphragm from flow in the proximal descending aorta, which was calculated from the sum of the arterial ductal flow and aortic isthmus flow. Aortic isthmus flow was calculated as the ascending aorta minus superior caval vein flow. The flow measurements were indexed to the foetal weight, based on segmentation of the foetus from a three-dimensional steady state-free precession acquisition, as previously described.^9^ Inter-observer variability for the flow measurements made in both groups was assessed by comparing the flow measurements made by two observers who were blinded to each other’s results using Pearson’s correlation and a Bland Altman plot.

All subjects were followed up to 1 year of age when a chart review was conducted and information about surgical procedures, death, or serious morbidity was collected.

### Statistical methods

The foetal flows are expressed in ml/min and indexed to the foetal weight in kg. Foetal flows are also expressed as percentages of the combined ventricular output, which is calculated from the sum of the ascending aorta and main pulmonary artery flows, plus 3% for coronary flow based on foetal lamb measurements.^11^ Owing to the small number of foetuses with transposition, a non-parametric statistical analysis was used to compare flows in transposition versus normal controls (two-tailed unpaired Mann-Whitney test). P-values<0.05 were considered significant.

## Results

### Foetal study

**Figure 1:**
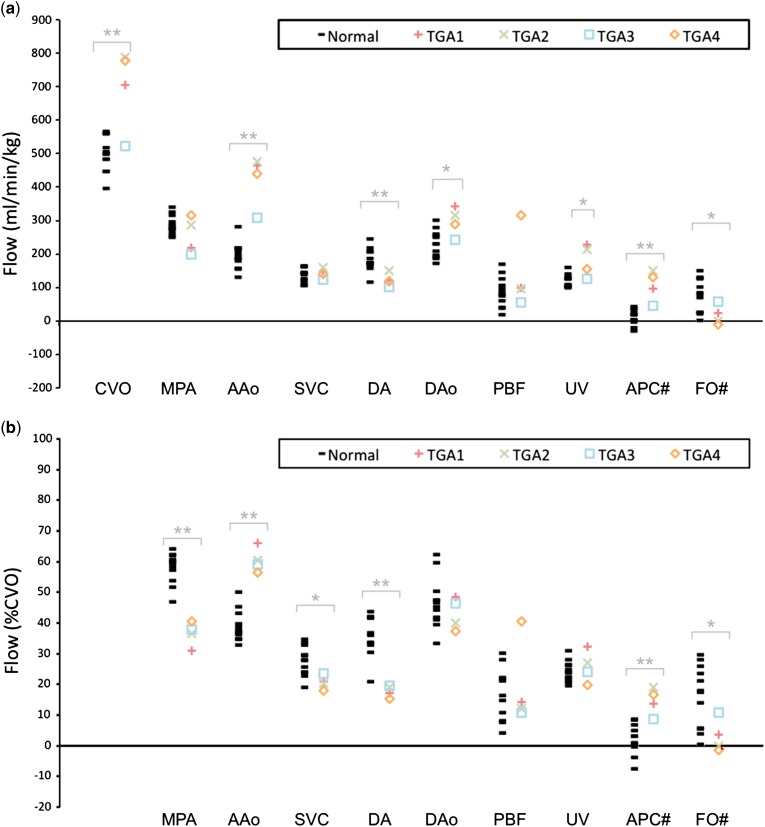
Flows in foetuses with transposition versus normal hearts. Normal foetal flows (black bars) are compared with transposition of the great arteries (TGA) (coloured symbols) in units of (***a***) ml/minute/kg, and (***b***) as percentage of combined ventricular output (CVO) (# denotes calculated flows). Significance level is indicated (*p<0.05, **p<0.01, ***p<0.001). AAo=ascending aorta; APC=aortopulmonary collateral; DA=arterial duct; DAo=descending aorta; FO=oval foramen; MPA=main pulmonary artery; PBF=pulmonary blood flow; SVC=superior caval vein; UV=umbilical vein.

Measurement of blood flow in all major foetal vessels was possible in all foetuses. An example of a phase-contrast MRI acquisition made in the descending aorta of a foetus with transposition at 37 weeks’ gestation is shown in Supplementary video S1. Flows are shown in Tables 1 and 2, and Figure 1. There was a high level of agreement between the two observers (r=0.99, p<0.001) with a negligible bias of −4.73±30.18 ml/minute across a range from 145 to 1150 ml/minute.

**Table 1:** Flows in 12 foetuses with normal hearts and four foetuses with transposition of the great arteries.

	GA (weeks)	EFW (kg)	CVO (ml/minute/kg)	MPA (ml/minute/kg)	AAo (ml/minute/kg)	SVC (ml/minute/kg)	DA (ml/minute/kg)	DAo (ml/minute/kg)	PBF (ml/minute/kg)	UV (ml/minute/kg)	FO (ml/minute/kg)*	APC (ml/minute/kg)*
Normal												
1	39	4.0	446	278	155	107	188	197	97	107	26	39
2	37	3.4	501	297	189	114	169	210	74	141	27	34
3	37	2.3	498	285	198	163	218	251	38	105	129	2
4	37	3.1	506	273	218	121	210	302	107	126	150	5
5	38	2.9	448	278	157	125	245	279	19	101	126	−2
6	34	2.0	566	341	208	107	173	256	125	125	22	18
7	36	2.8	561	263	281	165	117	230	170	110	132	3
8	37	3.3	519	315	188	145	174	173	146	160	3	44
9	36	3.0	485	251	219	163	159	191	79	128	103	24
10	38	2.8	397	255	130	138	166	188	88	104	71	−30
11	37	3.4	560	326	217	144	206	261	60	134	79	18
12	40	4.2	485	290	180	144	175	230	40	100	84	−19
Median	37	3.1	499	282	194	141	175	230	84	118	82	12
TGA												
1	35	2.6	705	219	465	148	122	342	100	228	25	97
2	38	3.1	789	288	477	161	151	316	96	213	0	151
3	38	3.6	522	198	308	123	102	242	56	125	57	45
4	37	2.4	778	315	440	140	120	290	315	155	−10	130
Median	38	2.9	742	254	453	144	121	303	98	184	13	114
p**			0.007	0.39	0.001	0.66	0.008	0.03	0.52	0.03	0.003	0.001

AAo=ascending aorta; APC=aorto-pulmonary collateral; CVO=combined ventricular output; DA=ductus arteriosus; DAo=descending aorta; EFW=estimated foetal weight; FO=foramen ovale; GA=gestational age; MPA=main pulmonary artery; PBF=pulmonary blood flow (sum of branch pulmonary arteries); SVC=superior vena cava; TGA=transposition of the great arteries; UV=umbilical vein Flows were measured by phase-contrast cardiovascular magnetic resonance with metric optimised gating and indexed by weight

* Calculated values

** Two-tailed unpaired Mann-Whitney test

**Table 2:** Flows in 12 foetuses with normal hearts and four foetuses with transposition of the great arteries.

	MPA (% cvo)	AAo (% cvo)	SVC (% cvo)	DA (% cvo)	Dao (% cvo)	PBF (% cvo)	UV (% cvo)	FO (% cvo)^*^	APC (% cvo)^*^
Normal (n=12)									
Median	60	37	28	36	46	19	24	18	2
Minimum/maximum	47–64	33–50	19–35	21–55	33–62	4–30	20–31	1–30	−8 to 9
TGA									
1	31	66	21	17	49	14	32	4	14
2	37	60	20	19	40	12	27	0	19
3	38	59	24	20	46	11	24	11	9
4	40	57	18	15	37	40	20	−1	17
Median	37	60	21	18	43	13	25	2	15
p**	0.001	0.001	0.02	0.001	0.52	0.98	0.63	0.02	0.002

AAo=ascending aorta; APC=aortopulmonary collateral; CVO= combined ventricular output; DA=ductus arteriosus; DAo=descending aorta; FO=foramen ovale; MPA=main pulmonary artery; PBF=pulmonary blood flow; SVC=superior vena cava; TGA=transposition of the great arteries; UV=umbilical vein Flows were measured by phase-contrast cardiovascular magnetic resonance with metric optimised gating and expressed as a percentage of the combined ventricular volume

* Calculated values

** Two-tailed unpaired Mann-Whitney test

The median combined ventricular output (p=0.001), ascending aortic (p=0.01), descending aortic (p=0.03), and umbilical venous (p=0.03) flows were increased in foetuses with transposition compared with normals. Median flows in the arterial duct (p<0.01) and oval foramen (p=0.03) were significantly lower in foetuses with transposition. When flow was expressed as a percentage of the combined ventricular output, we also found significantly lower median flows in the arterial duct and across the oval foramen, and higher flow in the ascending aorta in transposition foetuses. In addition, we found significantly reduced flow in the main pulmonary artery (p<0.001) and superior caval vein (p=0.02) in transposition. There was no significant difference between descending aortic and umbilical venous flow between the two groups in terms of percentage of the combined ventricular output. There was no significant difference in pulmonary blood flow in ml/min/kg or as a percentage of combined ventricular output between the two groups.

At 114 ml/minute/kg or 15% of the combined ventricular output, the median aorto-pulmonary collateral flow was significantly higher in the transposition group than the median aorto-pulmonary collateral flow of 11.5 ml/minute/kg or 2% of the combined ventricular output in the normal group (p=0.001).

### Clinical outcomes

Each of the four transposition foetuses underwent emergency balloon atrial septostomy at birth followed by an arterial switch operation in the neonatal period. Of the four patients, three made rapid progress and had no residual lesions or significant morbidity at 1 year of age. Transposition subject 2 continued to struggle in the postoperative period and was found to have pulmonary hypertension. A cardiac catheterisation at 6 weeks of age revealed a pulmonary vascular resistance of 25 Wood units∙m^2^ and the patient died of right ventricular failure 6 months later. The aortogram performed during this catheterisation confirmed the presence of enlarged bronchial arteries, shown in Supplementary video S2.

## Discussion

It has been suggested that the normal streaming of well-oxygenated blood from the umbilical vein across the oval foramen would result in increased pulmonary blood flow and oxygen mediated arterial ductal constriction in foetuses with transposition, because in transposition the left ventricle is connected to the pulmonary artery.^5^ In foetal lambs, a small increase in arterial oxygen saturation resulted in a threefold increase in pulmonary blood flow and increase in left atrial pressure.^12^ The increased pulmonary venous return associated with the drop in pulmonary vascular resistance resulting from higher oxygen saturations in the pulmonary arteries might help to explain the reduced oval foramen shunting that we observed in transposition foetuses. Reduced arterial ductal flow in transposition compared with normals may be a consequence of the lower output of the left ventricle than the right ventricle in the normal foetal circulation.^9^ ^,^ ^11^ The dominance of the right ventricular contribution to the combined ventricular output is likely to reflect greater preload to the right heart in the foetal circulation, and this may explain the increased ascending aortic flow we found in transposition foetuses, where the right ventricle supplies the ascending aorta.

**Figure 2:**
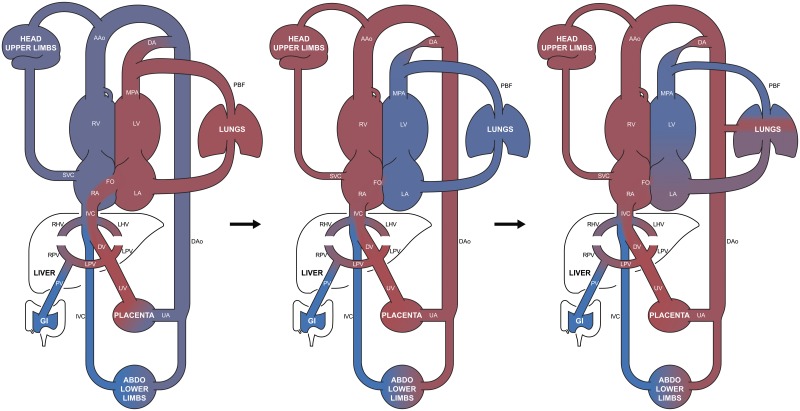
Potential mechanism for development of foetal pulmonary vascular disease in transposition of the great arteries. Initial pulmonary vasodilation because of streaming of oxygen from the umbilical vein (UV) to the pulmonary arteries results in oval foramen (FO) restriction and arterial ductal (DA) constriction, producing “isolation” and hypoxia of the pulmonary circulation, which in turn drives increased aorto-pulmonary collateral (APC) flow. AAo=ascending aorta; DAo=descending aorta; GI=gastrointestinal; IVC=inferior caval vein; LA=left atrium; LHV=left hepatic vein; LPV=left portal vein; LV=left ventricle; MPA=main pulmonary artery; PBF=pulmonary blood flow; RA=right atrium; RHV=right hepatic vein; RPV=right portal vein; RV=right ventricle; SVC=superior caval vein; UA=umbilical arteries.

Figure 2 depicts our hypothesis regarding the development of foetal pulmonary vascular disease in transposition. In the initial phase, there is pulmonary vasodilation resulting from the discordant ventriculo-arterial connections, with richly oxygenated blood from the umbilical vein streamed to the left heart and pulmonary arteries. However, the high pulmonary blood flow could then reduce the oval foramen shunt and the relatively low volume and high oxygen content of the blood now crossing the arterial duct could result in ductal narrowing. With reduced oval foramen and arterial duct shunts, the same blood would circulate between the lungs and left heart, with newly oxygenated blood from the placenta bypassing the left heart and passing directly to the systemic circulation. Under these circumstances, the blood entering the pulmonary circulation could become severely hypoxemic, as shown in the second phase of Figure 2. This is not unlike the postnatal situation in transposition, where there is frequently systemic hypoxia because of limited mixing between the systemic and pulmonary circulations. However, in the foetus, the pulmonary side would become cyanosed, as in foetal life the site of gaseous exchange is the placenta and not the lungs. The lower superior caval vein flow as a percentage of the combined ventricular output found in these foetuses with transposition could reflect a high oxygen content of the blood in the ascending aorta, the converse of the cerebral vasodilation known to occur during hypoxia as part of the brain-sparing response,^13^ as shown in the third phase of Figure 2. In a final step, the resulting gradient between the descending aorta and pulmonary arterial blood oxygen content could drive the overdevelopment of the bronchial circulation that is frequently present in newborns with transposition, therefore exposing the distal pulmonary arteries to increased blood pressure.

Previous studies have found evidence of increased aorto-pulmonary collateral blood flow in transposition. One series found enlarged bronchial arteries in 46% of infants with transposition.^14^ Increased in utero aorto-pulmonary collateral flow has also been demonstrated in transposition by echocardiography.^15^ Furthermore, the presence of enlarged bronchial arteries in infants with transposition has been associated with the early development of pulmonary vascular disease.^16^ Although increased aorto-pulmonary collateral flow may improve the oxygen content of pulmonary arterial blood in foetuses with transposition, it may expose the small pulmonary arteries to increased blood pressure. Ductal constriction, a known cause of foetal pulmonary vascular disease,^17^ may also elevate pulmonary pressures. Ductal constriction has been demonstrated in foetuses with transposition who went on to present with signs of pulmonary hypertension at birth.^3^ On the basis of our observations, the foetal pulmonary arteries in transposition could potentially be exposed to a combination of hypoxia and hypertension. This combination is known to cause pulmonary vascular disease in postnatal patients with congenital heart disease.^11^ Furthermore, hypoxia and ductal constriction both cause pulmonary vascular disease in foetal rats, a combination of the two results in the most severe pulmonary hypertensive changes.^18^

In our group of foetuses with transposition, the foetus with the lowest oval foramen shunt and highest aorto-pulmonary collateral flow developed fatal pulmonary vascular disease. The other three foetuses with transposition with good outcomes also showed reduced oval foramen and arterial ductal shunts and increased aorto-pulmonary collateral flow. However, none of these foetuses went on to develop pulmonary vascular disease, suggesting that additional circulatory or biological factors are likely to contribute to the development of pulmonary vascular disease in these patients. However, in keeping with another recent echocardiographic study,^19^ our measurements provide further evidence for an association between requirement of urgent atrial septostomy at birth and reduced shunting at the oval foramen and arterial ductal flow in foetuses with transposition. This is important because the accurate identification of foetuses with transposition at risk of developing pulmonary vascular disease could guide the timing of delivery and provide a potential target for foetal treatment. For example, improved mixing between the systemic and pulmonary circulations could potentially be encouraged by foetal interventions to open the atrial septum. These have been used to good effect in the setting of hypoplastic left heart syndrome with an intact or highly restrictive atrial septum in an attempt to halt the progression of pulmonary vascular disease resulting from pulmonary venous obstruction in utero and avoid the severe neonatal hypoxia that otherwise occurs.^20^ ^,^ ^21^ Such an approach has been suggested in the setting of transposition with an intact atrial septum,^22^ although we are not aware this has ever been attempted. It has also been suggested that occlusion of the ductus venosus in foetuses with transposition might prevent the pulmonary vasodilation, which may initiate the pathway towards pulmonary vascular disease in utero.^5^

We are uncertain of the reason for the higher combined ventricular output seen in the transposition foetuses compared with controls in this study. The increased cardiac output would appear to be the cause of the high descending aortic and umbilical venous flow, as these were normal proportions of the combined ventricular output. The increased combined ventricular output in transposition is in contrast with the 20% reduction we found in foetuses with left-sided obstructive congenital heart disease,^10^ but similar to the increased combined ventricular output that has been observed in foetuses with a drop in afterload associated with congenital absence of the ductus venous associated with anomalous drainage of the umbilical vein.^23^ It is possible that the expected streaming of oxygenated blood across the oval foramen could result in an increase in combined ventricular output associated with a reduction in afterload for both ventricles resulting from oxygen-mediated pulmonary and cerebral vasodilation. Regardless of the cause, the observation of high flow and potentially high oxygen delivery to the lower body is in keeping with the macrosomia previously observed in newborns with transposition.^24^

### Study limitations

The main limitation of this study is the small number of subjects. Although our small group of foetuses with transposition had similar patterns of distribution of blood flow in utero, only one developed severe irreversible pulmonary vascular disease. We believe there are other patterns of flow distribution in foetuses with transposition, and any potential clinical significance of these will only become apparent with larger numbers. Although the conclusions about the oxygen content of the blood in the systemic and pulmonary circulation in our subjects are the logical development of our own and previous observations, they remain speculative. However, the development of foetal blood oxygen-level-dependent MRI^25^ and foetal magnetic resonance oximetry^26^ offer a potential approach for investigating this hypothesis. Furthermore, we believe the novelty of this approach and the significant clinical outcome of one of our preliminary subjects warrant the reporting of this initial sample.

## Conclusion

In this study comparing the distribution of blood flow in a small group of foetuses with transposition with controls, we found reduced shunting across the oval foramen and arterial duct and increased aorto-pulmonary collateral flow in foetuses with transposition. One subject developed fatal pulmonary vascular disease following the arterial switch, raising the possibility that poor mixing between the systemic and pulmonary circulations in utero could be associated with early changes in pulmonary vascular development.

## Supplementary material

To view supplementary material for this article, please visit http://dx.doi.org/10.1017/S1047951114000870.
